# Co-grafts of Human Embryonic Stem Cell Derived Retina Organoids and Retinal Pigment Epithelium for Retinal Reconstruction in Immunodeficient Retinal Degenerate Royal College of Surgeons Rats

**DOI:** 10.3389/fnins.2021.752958

**Published:** 2021-10-26

**Authors:** Biju B. Thomas, Bin Lin, Juan Carlos Martinez-Camarillo, Danhong Zhu, Bryce T. McLelland, Gabriel Nistor, Hans S. Keirstead, Mark S. Humayun, Magdalene J. Seiler

**Affiliations:** ^1^Department of Ophthalmology, USC Roski Eye Institute, University of Southern California, Los Angeles, CA, United States; ^2^USC Ginsburg Institute for Biomedical Therapeutics, University of Southern California, Los Angeles, CA, United States; ^3^Department of Physical Medicine and Rehabilitation, University of California, Irvine, Irvine, CA, United States; ^4^Stem Cell Research Center, University of California, Irvine, Irvine, CA, United States; ^5^Department of Pathology, Keck School of Medicine, University of Southern California, Los Angeles, CA, United States; ^6^AIVITA Biomedical Inc., Irvine, CA, United States; ^7^Department of Ophthalmology, University of California, Irvine, Irvine, CA, United States; ^8^Department of Anatomy and Neurobiology, University of California, Irvine, Irvine, CA, United States

**Keywords:** retinal transplantation, retinal degeneration, human embryonic stem cells (hESCs), tissue engineering, vision testing

## Abstract

End-stage age-related macular degeneration (AMD) and retinitis pigmentosa (RP) are two major retinal degenerative (RD) conditions that result in irreversible vision loss. Permanent eye damage can also occur in battlefields or due to accidents. This suggests there is an unmet need for developing effective strategies for treating permanent retinal damages. In previous studies, co-grafted sheets of fetal retina with its retinal pigment epithelium (RPE) have demonstrated vision improvement in rat retinal disease models and in patients, but this has not yet been attempted with stem-cell derived tissue. Here we demonstrate a cellular therapy for irreversible retinal eye injuries using a “total retina patch” consisting of retinal photoreceptor progenitor sheets and healthy RPE cells on an artificial Bruch’s membrane (BM). For this, retina organoids (ROs) (cultured in suspension) and polarized RPE sheets (cultured on an ultrathin parylene substrate) were made into a co-graft using bio-adhesives [gelatin, growth factor-reduced matrigel, and medium viscosity (MVG) alginate]. *In vivo* transplantation experiments were conducted in immunodeficient Royal College of Surgeons (RCS) rats at advanced stages of retinal degeneration. Structural reconstruction of the severely damaged retina was observed based on histological assessments and optical coherence tomography (OCT) imaging. Visual functional assessments were conducted by optokinetic behavioral testing and superior colliculus electrophysiology. Long-term survival of the co-graft in the rat subretinal space and improvement in visual function were observed. Immunohistochemistry showed that co-grafts grew, generated new photoreceptors and developed neuronal processes that were integrated into the host retina. This novel approach can be considered as a new therapy for complete replacement of a degenerated retina.

## Introduction

Age-related macular degeneration (AMD) and retinitis pigmentosa (RP) lead to a profound loss of vision in millions worldwide. Many of these patients require replacement of both retinal pigment epithelium (RPE) and photoreceptors (PRs). During the early stages of retinal degeneration (RD), when most of the photoreceptors are preserved in the retina, damaged photoreceptors can be rescued by treatments, such as gene therapy (Bordet and Behar-Cohen, 2019; Maeda et al., 2019) and trophic factors (LaVail, 2005; Kolomeyer and Zarbin, 2014; Yang J. Y. et al., 2021); cell transplantation has also demonstrated positive effects which are considered to be mostly due to the introduction of neuroprotective growth factors released into the retina (Jayakody et al., 2015; Lingam et al., 2021; Nair and Thomas, 2021; Yang J. M. et al., 2021).

During advanced stages of the RD diseases, when most photoreceptors are degenerated or dysfunctional, neuroprotective approaches will not be very effective and cell replacement therapy has great potential for visual restoration, although retinal prosthesis (Yue et al., 2016; Flores et al., 2019; Prevot et al., 2020; Shim et al., 2020) and gene therapy (Yanik et al., 2016; McClements et al., 2020; Orlans et al., 2021) have shown some promising results. Cell-based techniques for the replacement of RPE, photoreceptors, and other inner retinal cells have been a major focus for various research groups (Zarbin, 2016; Jin et al., 2019; Zerti et al., 2021; Zhang et al., 2021). Clinical trials on cell-based therapies (including RPE and photoreceptor precursors) have been initiated at various facilities (Zarbin, 2016; Wang et al., 2020; Sharma and Jaganathan, 2021). In RPE transplantation experiments, while other investigators have proposed injecting suspensions of human embryonic stem cell (hESC)-RPE cells into the subretinal space (Schwartz et al., 2016), we have shown that hESC can be differentiated into highly polarized cells with molecular and functional attributes similar to that of the normal RPE (Thomas et al., 2016). For this, RPE cells were cultured on an ultrathin parylene substrate that can act as an artificial Bruch’s membrane (BM) (Lu et al., 2012). The product, termed CPCB-RPE1 implant, is being evaluated in an Food and Drug Administration (FDA)-approved phase1/2a clinical trial (NCT 02590692) for dry AMD (Kashani et al., 2018). Retinal sheet transplantation is another approach aimed at replacing the lost photoreceptors. This approach is promising for treating advanced degenerative conditions where replacement of photoreceptors is required (Zarbin, 2016). Transplantation studies based on grafting sheets of fetal-derived neural retinal progenitor cells with its RPE have been demonstrated in animal models (Aramant et al., 1999; Thomas et al., 2006; Seiler and Aramant, 2012; Lin et al., 2018) and humans (Radtke et al., 2008). However, ethical issues, immunological concerns and limited availability of fetal retina are major constrains for implementing this approach. To overcome these issues, 3D retinal sheets [retina organoids (ROs)] derived from stem cells are being tested for cell replacement therapies (Assawachananont et al., 2014; Zhong et al., 2014; Mandai et al., 2017; McLelland et al., 2018; Lin et al., 2020; Singh and Nasonkin, 2020). We have shown that hESC-ROs, transplanted into immunodeficient RD rats, showed good survival, matured into photoreceptors and inner retinal neurons and showed signs of integration and restoration of visual function (McLelland et al., 2018; Lin et al., 2020). Other groups have shown that transplanted cone precursors derived from ROs can improve vision in advanced RD (Ribeiro et al., 2021; Zerti et al., 2021). However, a well-defined RPE monolayer has been never observed in ROs (Llonch et al., 2018; Singh et al., 2018, 2021; Jin et al., 2019), presumably due to the difference in culture conditions, which is currently a limiting factor for hESC-derived retinal sheet transplantation.

Degeneration/dysregulation of RPE, a supportive monolayer of cells underlying the photoreceptors, is commonly seen in patients with RD diseases, such as AMD. It is suggestive that a combination therapy involving both PRs and RPE is a requisite for curing such irreversible retinal damages. Apart from RD diseases, accidents including laser-induced damages in the eye can cause irreversible retinal injuries (Liang et al., 2017; Commiskey et al., 2019). The studies showing an increase in integration of the stem cell-derived grafts in a laser damaged host retina supports our hypothesis that the co-graft technique can be employed for structural and functional reconstruction of a permanently damaged retina (Diniz et al., 2013). To demonstrate this technique, in the current study, a co-graft approach was used for transplantation experiments. The co-grafts were made of ROs and a hESC derived RPE (hESC-RPE) monolayer cultured on ultrathin parylene that can act as an artificial BM (Lu et al., 2012; Diniz et al., 2013; Thomas et al., 2016). The RPE component was adhered to RO sheets using a suitable bio-adhesive and transplanted into the rat eyes as a composite graft. Since the RPE monolayer is integral to maintaining healthy photoreceptors; and interacts with the photoreceptors in the phototransduction cycle (Bird, 1995), the presence of a healthy polarized RPE monolayer and structural support from intact and healthy BM will provide the suitable microenvironment for regenerating the RO sheets. Further, the presence of a supporting matrix can protect the co-graft from pathological BM. This can also prevent BM abnormalities from unfavorably altering the transplanted cells’ behavior. Another group has also published a cotransplantation approach (Mitrousis et al., 2020). Their approach was to mix hESC-RPE and mouse NRL-GFP photoreceptor progenitors in a hydrogel matrix, and transplant these to the subretinal space of immunosuppressed mice pre-treated with sodium iodate. Studies extended only up to 7 weeks post-transplantation. There was some improvement in scotopic visual acuity and light avoidance tests, and a slight improvement in ERG.

The current study used the immunodeficient (nude) Royal College of Surgeons (RCS) rats as the RD rat model, which were created by crossing RCS rats with the defective Mer tyrosine kinase (MerTK) gene with athymic nude rats (Hsd:RH-Foxn1mu, mutation in the foxn1 gene; no T cells, but presence of natural killer cells) (Thomas et al., 2018). The MerTK receptor mutation abolishes internalization of photoreceptor outer segments (OSs) (D’Cruz et al., 2000). Because of the RPE dysfunction caused by the deletion of MerTK receptor in the RCS rats, photoreceptors degenerate slowly. Almost complete loss of photoreceptors and visual function is observed around 90 days of age (Nandrot and Dufour, 2010; Thomas et al., 2018).

Although the eye is considered as a relatively immunoprivileged organ, immunosuppression is still recommended for xenografts (Warfvinge et al., 2006; Ilmarinen et al., 2015). However, immunosuppression is labor intensive, may cause additional pain to the animals, and adversely alters visual behavioral and electrophysiological testing (Lu et al., 2009; Anderson et al., 2011; Kamao et al., 2014; Cooper et al., 2016). Because nude RCS rats do not reject xenografts due to lack of T-cells (Thomas et al., 2018), there is no need for immunosuppression.

In our current study, due to the difference in culture conditions of RPE and ROs, RPE and ROs were generated separately and combined using different bio-adhesives before transplantation into nude RCS rats. The longtime survival of co-graft and visual function improvement was studied using different methods.

## Materials and Methods

### Generation of Retina Organoid Sheets

Retina organoids were differentiated from two cell lines. The first one, the cone-rod homeobox gene-green fluorescent protein (CRX-GFP) hESC line (Collin et al., 2016), was maintained with TeSR E8 media on Vitronectin XF (Stemcell Technologies) coated plates. Cells were passaged every 4–5 days at ∼80% confluency using ReLeSR reagent (Stemcell Technologies) and 5 μM ROCK inhibitor to enhance cell survival. The media was changed daily. The CRX-GFP cell line is derived from H9 (NIH 0043) and was obtained at University of California, Irvine (UCI) through a material transfer agreement (MTA) with University of Newcastle. The second hESC cell line, CSC-14 (NIH 0284), was grown and expanded using a chemically defined and xeno-free custom formulated media (Irvine Scientific, Irvine, CA, United States) supplemented with low levels of bFGF and Activin-A. Cells were grown on thin Matrigel^TM^ (Corning, Corning, NY, United States) and passaged every 3–4 days at 1:6–1:10 splits using Collagenase IV digestion. ROs from both cell lines were generated using a protocol previously described (Zhong et al., 2014). Organoids from the CSC-14 line (obtained from AIVITA Biomedical, Irvine, CA, United States) were used for the initial gelatin and matrigel embedding experiments, whereas all the remaining alginate embedding experiments were performed with the CRX-GFP cell line (organoids differentiated at UCI).

Retina organoids were selected that contained an outer transparent layer and had developed a hollow spherical shape with a laminated structure, as seen by phase contrast and dissection microscope (see Supplementary Figure 1 in McLelland et al., 2018). Rectangular RO sheets (0.7–1.3 mm × 0.6 mm) were cut out from these structures for transplantation. Adherent RPE aggregates were dissected away (McLelland et al., 2018). Organoid dissection was performed immediately before assembling the co-graft and before transplantation.

### Flow Analysis

Retina organoids derived from the CRX-GFP H9 cell line were dissociated into a single cell suspension using papain enzyme (Worthington Biochemical) according to manufacturer’s instructions. Cells were analyzed for GFP expression at various timepoints using an ACEA NovoCyte Flow Cytometer. Cells were sorted on a BD FACSAria II. Flow plots were generated using FlowJo software.

### Quantitative PCR

**FIGURE 1:**
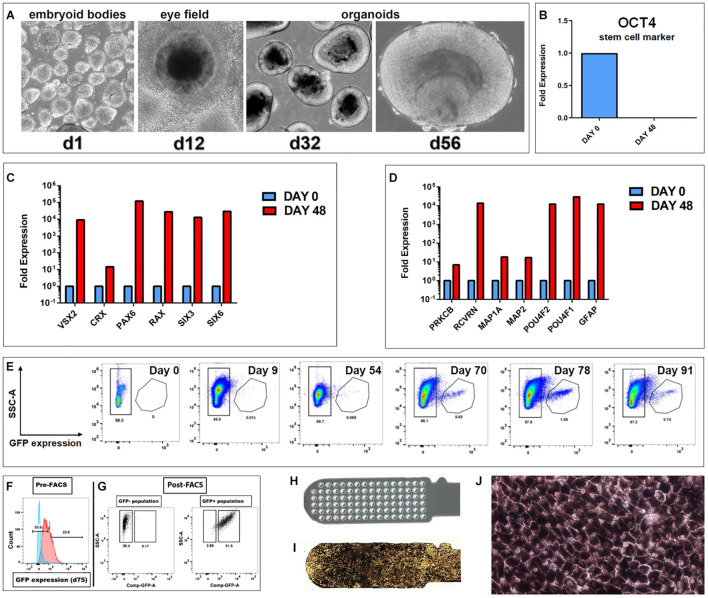
Generation of hESC derived retina organoids (CRX-GFP cell line) and RPE (H9 cell line). **(A)** Phase contrast images of organoid development up to day 56. **(B–D)** Progenitor gene expression analysis of CRX-GFP hESC-derived retina organoids (*n* = 2) and undifferentiated stem cells (*n* = 1). **(B)** OCT4 expression is absent in differentiated day 48 retina organoids. **(C)** Retina organoids (red) highly express genes indicative of primitive retina and photoreceptor progenitors. **(D)** Markers for mature, terminally differentiated retinal subpopulations. Retina organoids (red) contain markers for all the major retinal cell types, including photoreceptor progenitors, glia, ganglion cells, bipolar, horizontal, amacrine, and Müller cells. **(E)** Flow analysis shows that GFP expression is absent in the undifferentiated stem cells, early embryoid bodies, and young retina organoids. A distinct GFP+ population appears at day 70. Expression is highest day 78 and persists in the oldest analyzed day 91 organoids. **(F)** Examples of gates drawn used for FACS sorting of the GFP negative and GFP positive populations. **(G)** Post-FACS purities >90%. Cells were gated based on singlets, SSC vs. FSC profile, and if they were DAPI negative. Negative controls were undifferentiated CRX-GFP hESC (day 0). **(H)** Parylene only implant (no cells). **(I)** CPCB-RPE1 implant covered with hESC-RPE cells. **(J)** Enlargement of **(I)** to show details of hESC-RPE cells forming an epithelium (28–35 days culture).

Differentiated ROs were analyzed at day 56 (*n* = 2, each sample consisting of 4 organoids) and compared with undifferentiated CRX-GFP stem cells (*n* = 1). Each sample was run in duplicate. The genes analyzed in Figure 1 are listed in Table 1. The unique primers are QuantiTect commercial primers (Qiagen). RNA was isolated using TRIzol reagent (Qiagen), DNase I digested (Thermo Fisher, Waltham, MA, United States), and phenol:chloroform extraction (Thermo Fisher). cDNA was generated using the QuantiTect Reverse Transcription Kit (Qiagen). Amplification was performed using the QuantiFast SYBR green PCR master mix (Qiagen) and with the following cycling conditions: 95°C (10 min); followed by 40 cycles of 95°C (1 min), and 60°C (30 s). Ct values were determined using Viia7 RUO software (Thermo Fisher). Delta Ct values were calculated using RPL7 as the housekeeping gene. The mean Delta Ct value per gene was determined.

**TABLE 1:** List of analyzed genes.

Gene ID	Accession number	Official full name
CRX	NM_000554	Cone-rod homeobox
PAX6	NM_000280	Paired box 6
PRKCB	NM_002738	Protein kinase C, beta
RAX	NM_013435	Retina and anterior neural fold homeobox
RCVRN	NM_002903	Recoverin
SIX3	NM_005413	SIX homeobox 3
SIX6	NM_007374	SIX homeobox 6
VSX2	NM_182894	Visual system homeobox 2
MAP2	NM_002374	Microtubule-associated protein 2
POU4F2	NM_004575	POU class 4 homeobox 2
POU4F1	NM_006237	POU class 4 homeobox 1
GFAP	NM_002055	Glial fibrillary acidic protein
MAP2	NM_002374	Microtubule-associated protein 2

### Generation of Human Embryonic Stem Cell-Retinal Pigment Epithelium Sheets

The hESC-RPE implants for transplantation experiments were made based on our previously established methodology (Thomas et al., 2016). Briefly, hESCs (NIH registered H9 cell line, WiCell Research Institute, Inc., Madison, WI, United States) were cultured in mTeSR1 medium (Stemcell Technologies), and spontaneously differentiated into RPE cells in serum-free and xeno-free X-VIVO 10 medium (Lonza, Walkersville, MD, United States). The pigmented RPE-like cells were enriched by enzymatic treatment that selectively harvests pigmented cells (see below). The isolated RPE like cells were dissociated by TrypLE (Life Technologies, Grand Island, NY, United States) and cultured in human vitronectin (BD Biosciences) coated plates with X-VIVO 10 medium. Passage 3 hESC-RPE cells were used to seed on the ultrathin parylene membranes. The parylene substrates used consisted of an ultrathin membrane of parylene (0.30 μm thick) with a 6 μm thick supporting mesh (Lu et al., 2012). The cells on the parylene membranes were cultured in X-VIVO for 28–35 days with medium changes twice a week.

### Preparation of Co-grafts

Generation of co-grafts was tested using three different bio-adhesives [gelatin, growth factor-reduced matrigel, and medium viscosity (MVG) alginate]. RO sheets were cut into suitable sizes and adhered to RPE implants using a thin layer of bio-adhesive, coated on the surface of the RPE implant. Experiments with gelatin and matrigel were performed with CSC-14 derived ROs, whereas the alginate-embedding experiments were performed with CRX-GFP derived ROs.

Gelatin (Sigma, St. Louis) was initially tested at 1–5%. However, it was impossible to keep RO and RPE sheets together, and it was therefore abandoned.

Growth factor-reduced matrigel (1:2) (Fisher Scientific, Waltham MA, United States; or Corning, Corning, NY, United States) was tested next. Matrigel needs to be maintained cold and will gel at room temperature. Therefore, the stage for manipulating the tissues needed to remain cold. RO sheets were placed into a drop of matrigel (50 μL) which was then spread out. The matrigel was then allowed to polymerize for 5–10 min in an incubator before adding medium.

The MVG alginate (1%) (Pronova, Oslo, Norway) solution was prepared in media and remained liquid for tissue manipulation. As with matrigel, ROs were placed onto an RPE sheet in a small drop of alginate (1%). The drop was then spread out; and 100 μmol CaCl_2_ (in water) added to polymerize alginate. Media was added 2–5 min later. Prior to implantation, the composite graft was loaded into the nozzle of a custom implantation tool (Seiler and Aramant, 2012; Seiler et al., 2017; McLelland et al., 2018; Lin et al., 2020). In some experiments, the tissue was loaded into the nozzle in unpolymerized alginate, and the nozzle tip then dipped in CaCl_2_ for polymerization.

### Experimental Animals

For all experimental procedures, animals were treated in accordance with the NIH guidelines for the care and use of laboratory animals, the ARVO Statement for the Use of Animals in Ophthalmic and Vision Research, and under a protocol approved by the Institutional Animal Care and Use Committee of UCI (IACUC #2006-2698, and AUP-18-145), and of University of Southern California, Los Angeles (USC) (IACUC protocol # 21068). RCS (nude rat) transplant recipients were generated by pigmented dystrophic RCS rats (RCS-p+) with Hsd:RH-Foxn1rnu (mutation in the *foxn1* gene; no T-cells) rats (Thomas et al., 2018). Therefore, recipient rats have a mutant MerTK gene and a T-cell deficiency resulting in immunocompromised and retinal degenerate rats. To prevent infections of nude rats, rats were inspected daily, and cages changed under a laminar flow hood. Because nude rats do not produce tears, debris will accumulate under eyelids and result in infections. Therefore, eyes of nude rats were cleaned every 2 weeks under isoflurane anesthesia.

### Subretinal Implantation in Rats

Recipient immunodeficient RCS rats (P46-66, either sex) were randomized into age-matched non-surgery (*n* = 12), sham (*n* = 12), and transplant (co-graft, *n* = 30) cohorts. One group of co-graft surgeries (*n* = 18) was performed at USC, and initially tested at USC and then transferred to UCI for further tests; another group (*n* = 12) was performed and tested at UCI, with RPE sheets on parylene provided by USC. The same person (MS) was responsible for tissue preparation.

The animals were anesthetized with ketamine and xylazine (40–55 mg/kg Ket, 6–7.5 mg/kg Xyl), pupils dilated with 1% atropine eye drops (Akorn Pharmaceuticals, Lake Forest, IL, United States). Before anesthesia, rats received a subcutaneous injection of Ketoprofen (4 mg/kg) (Parsippany-Troy Hills, NJ, United States), and dexamethasone eye drops (Bausch & Lomb Inc., Rancho Cucamonga, CA, United States) to prevent eyelid swelling. The eye was disinfected with ophthalmic betadine (Alcon, Fort Worth, TX, United States). The non-surgical eye received artificial tears ointment (Akorn, Lake Forest, IL, United States) to keep the cornea hydrated. During the surgical procedure, the surgery eye was frequently treated with 0.5% tetracaine (Bausch & Lomb) and 0.1% dexamethasone eye drops (Bausch & Lomb). For recovery, rats were given a subcutaneous injection of Ringer saline solution and the analgesic Buprenex (0.03 mg/kg) i.p. (Reckitt Benckiser Pharmaceuticals, Richmond, VA, United States) for pain management. The surgical eye received additional treatment with betadine, followed by gentamycin/polymycin/bacitracin ointment (Bausch & Lomb).

Transplantation of retinal sheets has been previously described by our laboratory (Seiler et al., 2017; McLelland et al., 2018; Lin et al., 2020). Briefly, a small incision (∼1 mm) was made posterior to the pars plana, parallel to the limbus, followed by local mechanical retinal detachment. Donor retinal transplant tissue was delivered to the subretinal space of the left eye (LE) using the implantation instrument. Sham surgery consisted of placing the instrument into the subretinal space and injecting media alone. The incision was closed with 10-0 sutures. Rats were placed in a Thermocare (Thermocare, Paso Robles, CA, United States) incubator for recovery (or into a cage with a heating pad underneath).

### Spectral Domain Optical Coherence Tomography Imaging

Spectral domain optical coherence tomography (SD-OCT) imaging was used to document and monitor the transplant as it developed in the host retina. The general protocol was described previously (Seiler et al., 2017; McLelland et al., 2018). At USC, SD-OCT scans were performed using Spectralis© (Heidelberg Engineering, Inc.); at UCI, SD-OCT images of the retina were obtained using a Bioptigen Envisu R2200 Spectral Domain Ophthalmic Imaging System (Bioptigen, Research Triangle Park, NC, United States) after rats were anesthetized with ketamine/xylazine and the eyes were dilated with atropine. Transplanted rats (*n* = 29) were imaged either immediately after surgery (USC) or around 2 weeks after surgery (UCI), and then every 1–2 months after surgery, up to 9.5 months of age (7.7 months post-surgery). Rats with transplant misplacement into the vitreous, excessive surgical trauma such as optic nerve or corneal damage were excluded from further analysis after the first or second exam (*n* = 5). The last scan was scheduled as close as possible to the terminal experiment [superior colliculus (SC) recording]. Sham (*n* = 6) were imaged at approximately similar ages.

### Optokinetic Testing

Transplanted rats determined to have good-quality transplants (as assessed by SD-OCT), sham surgery, and non-surgery age matched controls (AMCs) underwent optokinetic testing (OKT) starting at the age of 4 months, corresponding to 2 months post-surgery. Two different systems were utilized for OKT at USC and UCI, respectively.

At USC, we have designed and built another OKT system, which is different from all the current commercially available ones and expected to show better visual function test (manuscript is in preparation). The OKT setup used two tablet screens to display the visual stimuli that consisted of high contrast black and white stripes generated using “OKN Stripes Visualization Web Application,” a freely available software. A clear plexiglass rat holder restrained the rat with its head continuously exposed to the OKT stimuli. Distance between the rat and the display screen was adjusted by moving the position of the rat holder. A micro camera attached to the top of the rat holder recorded the visual activity for later evaluation and quantitative assessment. The OKT responses at various spatial frequencies were assessed based on the presence or absence of a clear head-tracking response.

At UCI, OKT was measured with a commercially available system, by recording videos of optomotor responses to a virtual cylinder with alternating black and white vertical stripes (Optomotry, Cerebral Mechanics Inc., AB, Canada). The testing was described previously (Seiler et al., 2017; Lin et al., 2018). Rats were dark-adapted for at least 1 h prior to testing. Optomotor responses were recorded at six different spatial frequencies for 1 min per frequency by testers blinded to the experimental condition. Both the left and right eyes were tested by alternating the direction of the moving stripes. Two independent tests were performed at each time point, with at least 1-h time in between; one test going from lowest to highest frequency, and the other from highest to lowest frequency. The best visual acuity of the two tests was used for analysis. All tests were video recorded and evaluated off-line by two independent observers blinded to the experimental conditions. Any discrepancies between the two observers resulted in re-analysis of videos by a third observer, and data discussion before giving a final score, and prior to decoding the experimental condition.

### Superior Colliculus Electrophysiology

On selected rats, visual responses from the SC were recorded as previously described (Li et al., 2012; Seiler et al., 2017; Lin et al., 2018). During recording, the tester was blinded to the group allocation of the animal (age-matched non-surgery, sham, or transplant cohorts). After overnight dark-adaptation, responses from transplanted RCS nude rats (*n* = 8) were recorded between 5.9 and 7.7 months after surgery (age 7.6–9.5 months) and compared with responses from age-matched, non-transplanted RCS nude (*n* = 5), and sham rats (*n* = 6). A tungsten microelectrode (0.5 MΩ impedance; MicroProbe, Inc., Carlsbad, CA, United States) was used to record multi-unit electrical responses from 50 to 60 locations on the SC surface approximately 200–400 μm apart (ADInstruments, Inc., Colorado Springs, CO, United States). Light stimuli (20 ms duration) were delivered approximately 10-times at 10-s intervals at an intensity of 0.58 to −6.13 log cd/m^2^. When responses were found, the intensity of the light stimuli was reduced until there was no response to determine the threshold. Electrophysiological responses to the strongest light stimuli (stimulus level 0.58 log cd/m^2^) were quantified and formed into a map over the area of the SC that was analyzed. Any up or down deflection higher than the background recording in the 100 ms before stimulation was considered a response (spike). All spikes occurring 30–210 ms after the onset of the photic stimulus were counted. The sum was averaged across stimulus presentations. Analyses of the responses (spike counts and locations) were performed using a custom MATLAB program (MathWorks, Natick, MA, United States) (Seiler et al., 2017; Lin et al., 2018).

### Histology and Immunohistochemistry

After euthanasia with injection of anesthetic overdose [100 mg/kg Ket/20 mg/kg Xyl; or (at USC) euthasol], rats were perfusion-fixed with cold 4% paraformaldehyde in 0.1 M Na-phosphate buffer at 1–7.7 months post-surgery (transplanted rats, *n* = 17; sham surgeries, *n* = 6; age-matched controls, *n* = 5). Eye cups were dissected along the dorso-ventral axis, infiltrated overnight in 30% sucrose before embedding in Tissue-Tek O.C.T. compound and frozen using −60°C isopentane on dry ice. Serial 10 μm cryostat sections were cut and stored at −20°C. Every fifth slide was stained using hematoxylin and eosin (H&E) and analyzed for the presence of donor tissue in the subretinal space of the RCS host. H&E-stained slides were imaged on an Olympus BXH10 using an Infinity 3-1U camera. For immunofluorescence (IFA) and diaminobenzidine (DAB) analyses, cryostat sections underwent antigen retrieval at 70°C with Histo-VT One (Nacalai USA Inc., San Diego, CA, United States) and blocked for at least 30 min in 10% donkey serum or 20% horse serum (DAB). Primary antibodies are listed in Table 2. Primaries were left on sections overnight at 4°C, at specified concentrations. After several phosphate-buffered saline (PBS) washes, slides were incubated for at least 30 min at room temperature in fluorescent secondary antibodies, Alexa Fluor 488 donkey anti-rabbit IgG (H + L), and Rhodamine X donkey anti-mouse IgG (H + L) or Biotinylated conjugated secondary antibodies (dilution of 1:200–1:400) (Jackson ImmunoResearch, West Grove, PA, United States). Fluorescent sections were coverslipped using Vectashield mounting media (Vector Labs, Burlingame, CA, United States) with 5 μg/mL 4,6-diamidino-2-phenylindole (DAPIı). 3′3-Diaminobenzidine (DAB)-staining for Stem Cell 121 (SC121), Recoverin, cellular retinaldehyde binding protein (CRALBP), or Ku80-stained was also performed using an ABC kit (Vector Labs, Burlingame, CA, United States) and developed with DAB for up to 4 min and according to manufacturer’s instructions. Fluorescence was imaged using a Zeiss LSM700 confocal microscope (Zeiss, Oberkochen, Germany) taking tiled stacks of 5–8 μ thickness at 40× (selected images). Zen 2012 software (Zeiss, Oberkochen, Germany) was used to extract confocal images. 3D images were extracted separately for each channel and combined in Adobe Photoshop CS6 software (San Jose, CA, United States).

**TABLE 2:** List of primary antibodies.

Primary antibodies						
Antigen	Species	Specific for	Dilution	Supplier	Catalog #	RRID
Bestrophin	Mouse	RPE	1:200–1:500 (fl. Ab)	Chemicon/Millipore	MAB5466	AB_2064603
CRALBP	Rabbit	RPE and Muller cells	1:1K (fl. Ab)	Dr. John Saari (University of WA) (Saari et al., 1984)	N/A	AB_2314227
CRX (cone-rod homeobox gene)	Rabbit	Photoreceptors; cone bipolar cells	1:50 (fl. Ab)	Biorbyt	orb192904	N/A
RLBP1 (=CRALBP)	Rabbit	RPE and Muller cells	1:200 (fl. Ab)	Fitzgerald (North Acton, MA, United States)	70R-19908	N/A
Ku80	Rabbit	Human nuclei	1:400 (fl. Ab) 1:2K (ABC)	Abcam	ab80592	AB_1603758
PKCα	Mouse	Rod bipolar cells	1:200 (fl. Ab)	Stressgen	KAM-PK020	AB_1193543
Recoverin	Rabbit	Photoreceptors, cone bipolar cells	1:2K (fl. Ab) 1:10K (ABC)	Millipore	AB5585-I	AB_2253622
Rhodopsin (rho1D4)	Mouse	Rods	1:100 (fl. Ab) 1:10K (ABC)	Dr. Robert Molday, University of British Columbia (Molday and MacKenzie, 1983)	N/A	N/A
RPE 65	Mouse	RPE	1:100 (fl. Ab)	Novus	NB100-35	AB_350269
SC-121 (STEM121)	Mouse	Cytoplasm of human cells	1:2K (fl. Ab) 1:25K (ABC)	Stem Cell Inc. (Newark, CA, United States)	AB-121-U-050	AB_2632385

**Secondary antibodies**						

**Conjugate**	**Species**	**Specific for**	**Dilution**	**Supplier**	**Catalog #**	**RRID**

Alexa Fluor 488	Donkey	Rabbit IgG (H + L)	1:400	Jackson ImmunoResearch (West Grove, PA, United States)	711-545-152	AB_2313584
Rhodamine Red-X	Donkey	Rabbit IgG (H + L)	1:400	Jackson ImmunoResearch	711-295-152	AB_2340613
Biotin-SP	Donkey	Rabbit IgG (H + L)	1:200	Jackson ImmunoResearch	711-065-152	AB_2340593
Alexa Fluor 488	Donkey	Mouse IgG (H + L)	1:400	Jackson ImmunoResearch	715-545-150	AB_2340846
Rhodamine Red-X	Donkey	Mouse IgG (H + L)	1:400	Jackson ImmunoResearch	715-295-151	AB_2340832
Biotin-SP	Donkey	Mouse IgG (H + L)	1:200	Jackson ImmunoResearch	715-065-140	AB_2340783

### Experimental Design and Statistical Analysis

Rats (either sex) were randomized into age-matched non-surgery, sham, and transplant cohorts. Experimenters were blinded to the condition of the animal. For all statistical analyses, the significance level was calculated in GraphPad Prism software (GraphPad Software LLC, La Jolla, CA, United States) with paired and unpaired two-tailed *t*-tests using mean ± SEM. Paired *t*-tests were used for comparisons of left and right eyes of the same group, unpaired *t*-tests were used for comparisons between groups. Level of significance was set at 0.05.

## Results

### Generation and Characterization of Retina Organoids and Human Embryonic Stem Cell-Retinal Pigment Epithelium Sheets

As shown in Figure 1A, CRX-GFP hESCs differentiated into eye field then ROs. At day 56 (before transplantation), expression of the stem cell marker OCT4 was not detectable in the organoids (Figure 1B), showing that all stem cells had differentiated and matured. Differentiation into retinal progenitor cells was indicated by strong expression of genes of primitive retina (VSX2, CRX, PAX6, RAX, SIX3, and SIX6) (Figure 1C) and terminally differentiated retinal cell populations (PRKCB, RCVRN, MAP1A, MAP2, POU4F2, POU4F1, and GFAP) (Figure 1D). These include expression of markers for progenitors of photoreceptors, glia, ganglion cells, bipolar, horizontal, amacrine, and Müller cells, similar to what we have shown in previous publications for the CSC-14 cell line (McLelland et al., 2018; Lin et al., 2020) and for the CRX-GFP cell line (Xue et al., 2021). Flow analysis showed that GFP expression was absent in the undifferentiated stem cells, early embryoid bodies, and young ROs. A large GFP+ population appeared at day 70. Expression was highest day 78 and persisted in the oldest analyzed day 91 organoids (Figure 1E). Figure 1F shows the gates drawn used for fluorescent activated cell sorting (FACS) of GFP-positive and -negative cell populations. Post-FACS purities were greater than 90%. RO sheets at different days of differentiation were used for transplantation: days 28–30 (*n* = 4), days 50–59 (*n* = 19), and days 169–186 (*n* = 6). At early stages, they contained few GFP+ cells (day 54 plot) (Figure 1E). Similar, more extensive data have been published about the CSC-14-hESC-derived ROs (McLelland et al., 2018).

Human embryonic stem cell-retinal pigment epithelium were produced by seeding a suspension of RPE cells onto a vitronectin-coated parylene scaffold (Figure 1H) followed by *in vitro* culture until the time of transplantation (Figures 1I,J). Like other melanized cell types, RPE cells developed melanosome organelles, which are the intracellular sites of melanin biosynthesis. After approximately 2 weeks of culture, RPE exhibited cellular pigmentation, and cells gradually appeared darker by visual inspection with additional time in culture. The cells on the parylene membrane were cultured in X-VIVO for 28–35 days with medium changes twice a week, according to established methods (Lu et al., 2012; Thomas et al., 2016).

### Generation of Co-grafts for Subretinal Implantation

**FIGURE 2:**
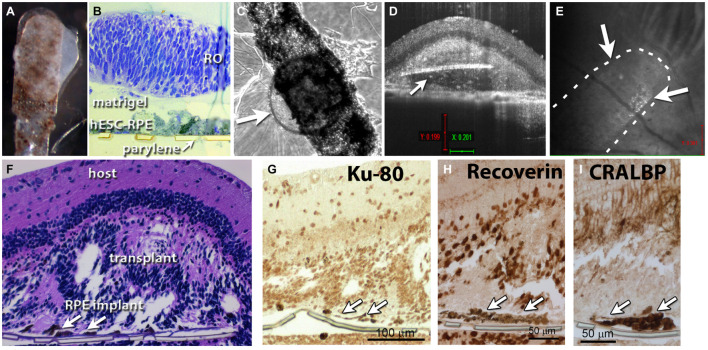
Retina organoid + RPE implant on matrigel. **(A)** RO and RPE embedded together using matrigel. **(B)** Plastic section of tissue fixed 1 h after matrigel embedding. **(C)** After co-culture for 4 days, arrow points to retina organoid. **(D)** Optical coherence tomography (OCT) B-scan image 12 days after transplantation of the co-graft into a rat eye **(A)**. Arrow indicates parylene implant. **(E)** OCT fundus image showing the co-graft placement in the subretinal area of an RCS rat; arrows point to co-graft. **(F)** Histology of the transplanted eye (hematoxylin–eosin staining). Arrows point to parylene implant. **(G)** Staining for human nuclei (Ku-80). **(H)** Recoverin: photoreceptors and cone bipolar cells. **(I)** Staining for cellular retinaldehyde binding protein (CRALBP: Müller glia and RPE).

**FIGURE 3:**
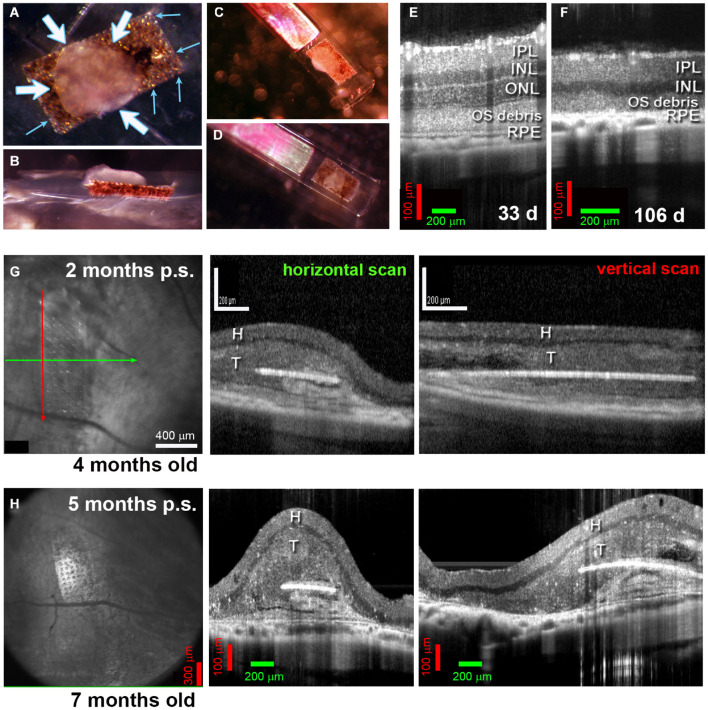
Alginate-embedded co-grafts. **(A,B)** Preparation of co-graft using MVG alginate. Retina organoid (RO) and RPE were adhered using 1% alginate. **(A)** View from top. Small blue arrows: RPE layer on the parylene membrane, white arrows: RO sheet adhered to the RPE layer. **(B)** Side view. **(C,D)** Examples of co-grafts loaded in nozzle of implantation instrument. **(E–H)** OCT B-scans. **(E)** 33-Day-old RCS retina (approximately 3 weeks before transplantation) still contains an outer nuclear layer (ONL). **(F)** At 106 d of age, the outer nuclear layer has completely disappeared. **(G)** Scans at 2 months post-surgery of transplant #1 in Figure 5 (Spectralis© Heidelberg Engineering, Inc., USC). Left panel: fundus image (maximum intensity projection). Arrows indicate position of horizontal B-scan (green) and vertical B-scan (red). Horizontal scan (center panel) and vertical scan (right panel) confirm the position of the co-graft in the subretinal space. **(H)** Corresponding scans of the same transplant at 5 months post-surgery (Bioptigen OCT, UCI). IPL, inner plexiform layer; INL, inner nuclear layer; ONL, outer nuclear layer; OS, outer segments; RPE, retinal pigment epithelium; H, host retina; T, transplant.

Generation of co-grafts was tested using different bio-adhesives. Initially, gelatin, matrigel, and alginate were selected for attaching the RO sheet over the RPE coated parylene implants. Gelatin is liquid above 37°C; and turns into gel when cooled down. However, it was very hard to handle it and retain the ROs with RPE as it would have required a heated stage. After many *in vitro* trials, we concluded that gelatin was not suitable and discontinued its use (data not shown). Co-grafts made using matrigel (*n* = 3) and MVG alginate (*n* = 27) were used for subretinal implantation experiments. Both bioadhesives allowed holding the RPE implant and RO sheet together (Figures 2, 3). Successful subretinal placement of the grafts was confirmed by optical coherence tomography (OCT) imaging. Good placement of the co-graft in the rat subretinal space was observed in one matrigel graft (*n* = 1/3) and 12 alginate (12/27) transplants. Based on the initial assessments, alginate showed better efficiency in maintaining co-graft integrity before and after implantation (Figure 3). Based on this, only MVG alginate was used for long-term transplantation experiments. We were unsuccessful in maintaining alginate co-grafts *in vitro*.

### Short-Term Assessments of Matrigel-Embedded Co-grafts

Figure 2A shows RO and RPE embedded together using matrigel shortly before transplantation. A plastic tissue section fixed 1 h after matrigel embedding showed a successfully produced co-graft, with some space separating the RO from the RPE sheet (Figure 2B). After co-culture for 4 days, co-grafts remained still healthy and together (arrow points to RO) (Figure 2C). However, with longer culture times, RPE migrated into the organoid and caused structural changes (data not shown). OCT B-scan images at 12 days after transplantation of a matrigel-embedded co-graft into a rat eye showed the co-graft placement in the subretinal (SR) area of an RCS rat (Figures 2D,E). However, as also seen later with alginate-embedded co-grafts, the RO had grown around the parylene RPE sheets (Figure 2D). Histology of the transplanted eye (H&E staining) confirmed the co-graft in the eye, and development of photoreceptors in rosettes (Figure 2F). Staining for human nuclei (Ku-80), recoverin (photoreceptors and cone bipolar cells), CRALBP (Müller glia and RPE) also confirmed the co-graft.

### Alginate-Embedded Co-grafts – *in vivo* Assessment of Morphology and Survival by Optical Coherence Tomography Imaging

Figures 3A–D shows the embedding procedure with alginate. Figures 3E,F shows the RD in immunodeficient RCS rats, with the outer nuclear layer (ONL) completely lost at 106 days (Figure 3F). OCT imaging conducted to assess the co-grafts revealed that the co-grafts survived in the subretinal space of immunodeficient RCS rats up to the end of the experimental term (up to 7.7 months post-surgery). The co-graft transplanted area appeared considerably thicker compared to the non-transplanted area (degenerated retina). The parylene membrane containing the RPE could be seen in maximum intensity fundus projections; and appeared as a white line in B-scans (Figures 3G,H).

### Optokinetic Testing Behavioral Assessments

**FIGURE 4:**
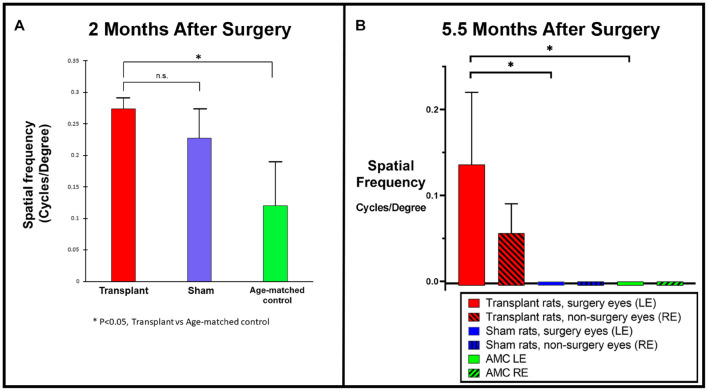
Optokinetic testing. **(A)** Improved visual acuity in the transplanted group revealed by OKT testing at 2 months post-surgery using OKT setup 1 (USC). A new setup that consisted of two tablet screens to display the OKT visual stimuli (high contrast black and white stripes) was used (manuscript under preparation). OKT responses at various spatial frequencies were assessed based on the presence or absence of head-tracking response. When tested at the 2 months post-surgery, higher visual acuity was observed in co-graft transplanted rats (*n* = 5) compared to sham (*n* = 4, not significant, unpaired *t*-test) and age-matched non-surgery control rats (*n* = 4, *p < 0.05, unpaired *t*-test). **(B)** OKT testing at 5.5 months post-surgery using OKT setup 2 (Cerebral Mechanics Inc.). Transplant surgery eyes (left, red bars) with co-graft show higher visual acuity compared to the fellow eyes (right, red bars with shading) of the same rats without transplants (*n* = 3, not significant), sham surgery (blue bars) (*n* = 6, *p < 0.05, unpaired *t*-test), and age-matched non-surgery controls (green bars) (*n* = 6, *p < 0.05, unpaired *t*-test). (The sham surgery and age-matched controls had consistently no OKT responses.) LE, left eye; RE, right eye; AMC, age-matched control.

Two different systems were utilized for OKT in UCI and USC, respectively. OKT data collected from USC showed improvement in co-graft transplanted rats (*n* = 5) compared to sham (*n* = 4, not significant) and age-matched non-surgery control rats (*n* = 4, *p* < 0.05) at 2 months post-surgery (Figure 4A). OKT data collected from UCI (from transplants performed at USC) at 5.5 months post-surgery (Figure 4B) also confirmed an improvement in the co-graft transplanted LEs compared to the right eyes of the same rats without surgery (*n* = 3, not significant due to low *n*). The improvement is significant compared to sham surgery rats (*n* = 6) and age-matched non-surgery controls (*n* = 6).

### Superior Colliculus Electrophysiology

**FIGURE 5:**
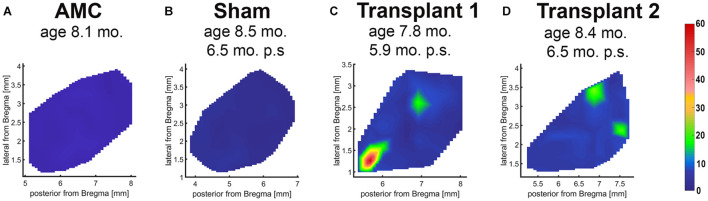
Superior colliculus recording. Spike count diagrams of responses from the surface of the SC after stimulation with full-field light flashes. No response was found in the entire SC area of non-surgery age-matched control **(A)** and sham rats **(B)**. Strong responses were found in some areas of the SC in 5 of 6 recorded transplanted rats (examples in **C,D**).

In the dystrophic RCS rats, RD progresses slowly. At the age of SC recording (260 ± 8.94 days-old), the rats showed complete loss of photoreceptors and visual function (Thomas et al., 2018; Lin et al., 2020). Electrophysiological mapping from the SC showed no response in any of the sham surgery rats (*n* = 6) or age-matched control animals (*n* = 5). Strong visual responses (spike activity) were recorded in the SC from co-graft transplanted RCS rats (*n* = 5) (Figure 5) at the age of 260 ± 8.94 days-old (204 ± 8.92 days after surgery). One rat even showed responses to very low levels of light stimulation at the scotopic level; the best light threshold was −1.31 log cd/m^2^ (Table 3).

**TABLE 3:** Superior colliculus recording data of RCS rats transplanted with co-graft or retina organoid.

Rat ID	dps at SC recording	Age (days)	Age of donor tissue (days)	Area with response (%)	Max spike count (average)	Response threshold log (cd/m^2^)	Number of rats with response	Total number (*N*)
Transplant 1	176	228	52	6.67	67.40	−0.15		
Transplant 2	195	253	53	4.00	26.09	−0.15		
Transplant 3	197	255	53	2.00	22.86	−0.15		
Transplant 4	224	278	186	2.33	26.15	−1.31		
Transplant 5	230	284	186d	10.00	30.83	−0.41		
Co-grafts to RCS rats (mean ± SEM)	204 ± 8.92	260 ± 8.94		5.00 ± 1.34	34.67 ± 7.41	−0.44 ± 0.20	5	5
Retina organoids to RCS rats*	236 ± 13.9	284 ± 13.8	30–65	17.07 ± 5.88	33.27 ± 5.97	−0.42 ± 0.29	7	14
Retina organoids to RD rats**	194 ± 16.88	220 ± 16.77	30–70	8.73 ± 3.44	25.8 ± 5.45	0.32 ± 0.09	9	13

*Data from previous work (Lin et al., 2020).

**Data from previous work (McLelland et al., 2018).

### Long-Term Morphological Assessment of Alginate-Embedded Co-graft Implanted Retinas

**FIGURE 6:**
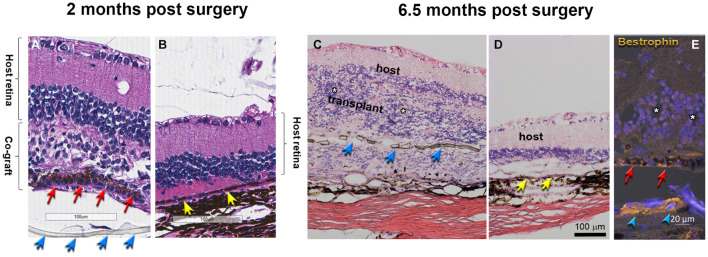
Transplant histology at 2 months **(A,B)** and 6.5 months **(C–E)** post-surgery. **(A,C)** Co-graft area. **(B,D)** Host retina outside co-graft. **(A–D)** H&E staining. White asterisks indicate the center of rosettes (spherical arrangement of photoreceptors and other retinal layers around a central lumen). The transplant in **(C)** has grown around the parylene RPE implant (transplant #3 in Table 3, 197 dps). **(E)** Bestrophin (gold) and DAPI (blue) shows RPE and parylene in transplant (#2 in Table 3, 195 dps). Blue arrows point to parylene. Red arrows point to hESC-RPE. Yellow arrows point to host RPE. The separation of tissue is processing artifact. Scale bars = 100 μm **(A–D)**; 20 μm **(E)**.

**FIGURE 7:**
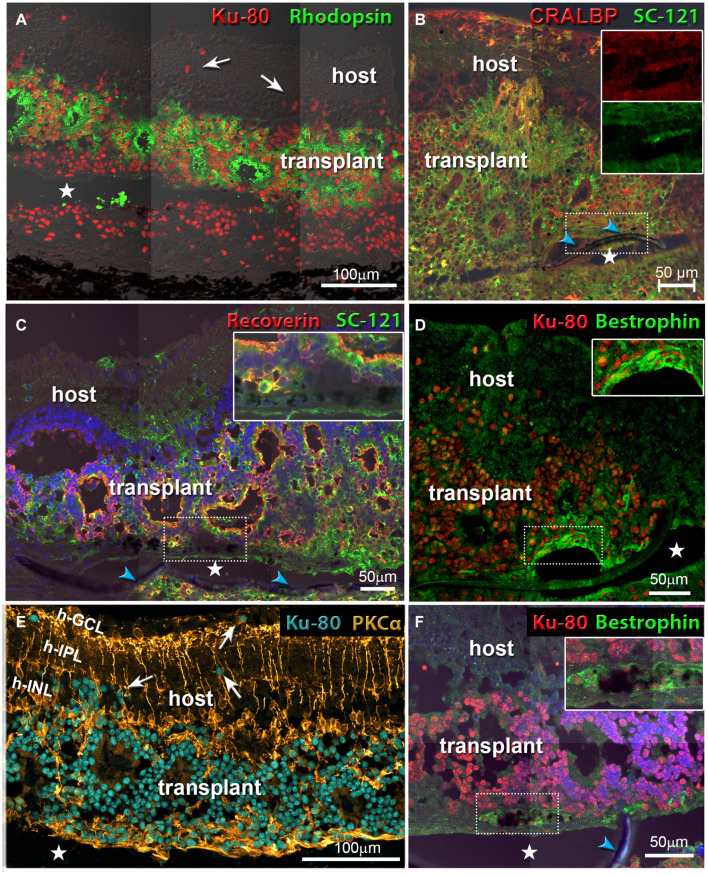
Co-graft transplants develop different cell types and migrate into the host retina. White arrows in **(A,E)** point to donor cells that have migrated into the host retina. Asterisks indicate tissue space created by processing artifact (parylene implant was located there but got lost during cutting). Blue arrowheads indicate the parylene membrane. **(A)** Rod photoreceptors (green) have developed outer segments in rosettes. Human nuclei are stained red. The transplant has grown around the parylene implant (transplant #1 in Table 3, 176 dps). **(B)** Müller glia cells and RPE stained for cellular retinaldehyde binding protein (CRALBP). Human cytoplasm is stained by SC-121 (green). The two inserts show that the RPE implant stains both for CRALBP and for SC-121 (transplant #3 in Table 3, 197 dps). **(C)** Recoverin (marker for photoreceptors and cone bipolar cells, red), and SC-121 (marker for human cytoplasm, green). Insert shows an enlargement of the implant-RO interface (not-SC-recorded transplant, 127 dps). **(D,F)** Combination of Bestrophin (RPE marker, green) and human nuclei (red). Inserts show enlarged area of implant. **(D)** Same transplant as in **(B)**. **(F)** Same transplant as in **(C)**. **(E)** Rod bipolar cells (PKCα, gold), human nuclei (turquoise). Same transplant as in **(A)**. Scale bars = 100 μm **(A,E)**; 50 μm **(B–D,F)**.

Morphological evaluation of the transplanted eyes was conducted by H&E staining and by immunohistochemistry. H&E images demonstrated reconstruction of severely damaged retina in the alginate-embedded co-graft implanted RCS rats. Assessments conducted at 2 months post-surgery showed good survival and signs of integration of the co-graft (Figure 6A) with the host retina (Figure 6B). Assessments at a later time point (6.5 months post-surgery) (Figures 6C–E) also revealed survival and maintenance of the transplant architecture, with transplant photoreceptors forming rosettes (Figures 6C,E, 7). Transplanted RPE cells were labeled by the RPE markers Bestrophin (Figures 6E, 7D,F) and RPE-65 (Supplementary Figure 1). Immunohistochemical labeling of the retinal sections showed that cells in the co-graft differentiate into rod photoreceptors and bipolar cells (Figures 7A,C,E). Some of these donor cells were found to be migrated into the host tissue (Figures 7A,E). Glial cell differentiation was also observed in several co-graft implanted retinas (Figure 7B).

## Discussion

Our study demonstrated that retina co-graft implantation is a feasible technique to reconstruct a severely damaged retina. We tested different bio-adhesive substrates for constructing the co-grafts. Our data showed that it is possible to make RPE and RO co-grafts using alginate as a bio-adhesive. Previous investigations demonstrated that alginate can be used for tissue engineering and other biomedical applications (Kuo and Ma, 2001; Espona-Noguera et al., 2018; Hontani et al., 2019; Yeo and Kim, 2020; Kapr et al., 2021). It’s application in wound healing, drug delivery, and tissue engineering is well established due its ability to retain structural similarity to the extracellular matrices in tissues (Lee and Mooney, 2012; Li et al., 2020). Matrigel is another established substrate used for tissue engineering research because it is easy-to-use, commercially available, and offers environments for three-dimensional (3D) cell culture that mimic native tissue (Lu et al., 2017; Ko et al., 2019; Capeling et al., 2020). However, our attempt to use it as a bio-adhesive for constructing the retinal co-graft was not successful. In our observation, matrigel failed to properly adhere the components of the co-graft together. In addition, our pilot *in vitro* analysis (data not shown) suggested that matrigel induced reorganization of the organoid structure. Alginate on the other hand showed better adherence and alignment of the implants.

The present study demonstrated that co-grafts made of RPE and ROs adhered together by MVG alginate can be successfully implanted into the subretinal space of RCS rats. These implants survived in the subretinal space for a substantial period of time (up to 7.7 months post-surgery). OCT imaging and histological assessments showed that retinal architecture was mostly maintained in the co-grafts implanted eyes suggesting that co-graft approach can be used for reconstructing a severely damaged retina.

In most of the previous investigations, either RPE alone (Thomas et al., 2016), photoreceptors as sheets (Seiler and Aramant, 2012; McLelland et al., 2018; Lin et al., 2020) or dissociated cells (Lu et al., 2009; Jayakody et al., 2015; Viringipurampeer et al., 2021; Zerti et al., 2021) were used for subretinal implantation. The RPE replacement approach is relatively less complicated due to limited requirements for integration and ability to perform phagocytic functions. Based on our hESC-RPE implantation studies in RCS rats (Thomas et al., 2016), although considerable photoreceptor rescue can be observed during earlier time points, only a sub-population of photoreceptors are preserved when assessed at a later time point (6-month post-implantation). This suggests that loss of photoreceptors takes place even after RPE replacement therapy, suggesting need for a replacement therapy involving both PR’s and RPE for achieving long-term benefits. Compared to RPE replacement, photoreceptor transplantation is challenging due to difficulty in developing faithful integration with the host neurons (Zarbin, 2016; Llonch et al., 2018; Singh and Nasonkin, 2020). In our recent investigation, hESC-ROs that are transplanted into immunodeficient RD rats survived, matured into photoreceptors and inner retinal neurons and showed promising indications of neuronal integration and restoration of visual function (McLelland et al., 2018). Despite the above positive effects, the transplanted ROs failed to maintain their lamination structure as a result of rosette formation (McLelland et al., 2018) as reported by other investigators (Assawachananont et al., 2014; Mandai et al., 2017). As formation of rosettes can adversely affect long-term survival, neuronal integration and visual functional contributions of ROs, an unmet need for further improvement of this technique is implied. Since the RPE monolayer is integral for maintenance of healthy photoreceptors; and provides the transducing interface for visual perception (Bird, 1995) with the photoreceptors, it is plausible that the presence of a healthy polarized RPE monolayer and structural support from an artificial BM (parylene substrate) will provide a suitable microenvironment for regeneration of the RO sheets. Considering the invasive nature of the retinal damage, a co-graft approach may be desirable for treating conditions where both photoreceptors and RPE are severely damaged. This can occur not only due to RD; there are accidents causing irreversible retinal injuries as in war fields (Roider et al., 1999; Harris et al., 2003; Commiskey et al., 2019). Exposure to laser beams is a major cause of irreversible damage to the photoreceptors, RPE, and choroid (Ben-Shlomo et al., 2006), eventually leading to permanent visual loss (Commiskey et al., 2019).

Visual functional assessments conducted in co-graft implanted rats showed considerable improvement based on OKT testing and SC electrophysiology.

In our previous work, ROs transplanted to RCS rats and RD rats also improved the visual function by SC recording (McLelland et al., 2018; Lin et al., 2020). As shown in Table 3, the improvement shown by area with response and max spike count was not significantly different from each other for the three types of transplants. However, transplanted RCS rats showed significantly lower light response thresholds in the co-grafted (*p* = 0.0017) group (this study) and in the RO-only transplanted group (*p* = 0.0228; Lin et al., 2020) indicating better improvement transplant effects in RCS rats than what has been reported in a previous study of RO transplant effects in *Rho S334ter line-3* RD rats (McLelland et al., 2018). There was no difference in visual responses between co-graft and RO transplanted RCS rats. These data may be caused by the difference of the two rat models: the RCS rat exhibits relatively slow RD; and some surviving host photoreceptors at the time of transplantation could be rescued by trophic effects from the transplants; while the RD rat is a model of fast RD where most photoreceptors died or were irreversibly damaged at the time of transplantation and could not be rescued. Interestingly, when compared with our previous studies (as shown in Table 3), a higher percentage of transplanted rats showed responses in the (A) co-graft group (this study), followed by (B) transplants to RD rats (McLelland et al., 2018) and (C) RO-only transplants to RCS rats (Lin et al., 2020) (A vs. C: *p* < 0.05. Not significant among comparison of other groups, which may be because of the low *N*). The data confirmed the need and advantage of co-grafting: the RCS rat is a retinal degenerative and RPE dysfunction model while RD rat is a retinal degenerative model with healthy RPE; thus the higher success rate of restoring light responses (tested by SC recording) by RO-only transplants in RD rats than in RCS rats suggested that besides RO, the RPE replacement is needed for RPE dysfunction/degenerative RD diseases; and this is confirmed by the highest response rate of co-grafts to RCS rats than seen with both RO-only transplants to RD rats and RO-only transplants to RCS rats.

As our study contains a relatively low number of animals, it is difficult to determine the mechanism of visual improvement. Transplants were performed at the age of 2 months when photoreceptors can no longer be rescued by RPE transplants and other rescue treatments (such as trophic factors and gene therapy) (Riera et al., 2016). Although there were remaining host photoreceptors seen, photoreceptors with OSs were only observed in the transplants.

Further, apparent neural integration between the transplant and host retina was observed although this does not prove synaptic connectivity yet. However, we have performed more detailed analysis in two previous papers about RO transplants which demonstrated co-localization of donor, host, and synaptic markers (McLelland et al., 2018; Lin et al., 2020). Furthermore, the co-grafting process needs to be optimized (testing other embedding substrates, organoid differentiation protocols, and donor ages) to reduce the occurrence of rosettes. Although further studies will be needed to show a significant advantage for co-graft implanted rats over the control groups (RO alone or RPE alone), it demonstrated strong evidence in favor of a co-graft transplantation approach.

While our study was performed in immunodeficient rats, some immunological reactions can still be an influential factor for graft survival integration, since *foxn1−/−* rats still have natural killer cells (Mehrotra et al., 2017). The significance of the co-graft implantation approach can be very relevant for clinical applications where reconstruction of a severely damaged retina is a requisite. It may be noted that experiments conducted using rat models have limitations due to the rats’ small eye size, morphological discrepancies, degree of injury manifestation and immunological activities. By demonstrating the “proof of concept” of co-graft implantation technique in an immunodeficient rat disease model, it is possible to translate our findings to treat advanced human RD diseases. It may be concluded that our novel tissue engineering-based co-graft approach will help to resolve a major hassle in the translational potential of a highly promising therapeutic product.

## Data Availability Statement

The original contributions presented in the study are included in the article/Supplementary Material, further inquiries can be directed to the corresponding author.

## Ethics Statement

The animal study was reviewed and approved by the Institutional Animal Care and Use Committee of UC Irvine (IACUC #2006-2698, and AUP-18-145) and Institutional Animal Care and Use Committee of USC (IACUC protocol #21068).

## Author Contributions

BT, BL, and MS: concept and design. BT, MS, GN, HK, and MH: financial support, administrative support, and provision of study material. BL, BT, DZ, JM-C, BM, and MS: collection and assembly of data. BT, BL, BM, and MS: data analysis and interpretation. BT, BL, MS, BM, GN, and HK: manuscript writing. BT, BL, JM-C, DZ, BM, GN, HK, MH, and MS: final approval of manuscript. All authors contributed to the article and approved the submitted version.

## Conflict of Interest

GN and HK are employed by AIVITA Biomedical Inc. HK is a board member and the CEO of AIVITA Biomedical Inc. MH is an investor, a consultant, and a board member of Regenerative Patch Technologies (RPT), and also holds an RPT patent. MS is a co-inventor on patents of Ocular Transplantation LLC. The remaining authors declare that the research was conducted in the absence of any commercial or financial relationships that could be construed as a potential conflict of interest.

## Publisher’s Note

All claims expressed in this article are solely those of the authors and do not necessarily represent those of their affiliated organizations, or those of the publisher, the editors and the reviewers. Any product that may be evaluated in this article, or claim that may be made by its manufacturer, is not guaranteed or endorsed by the publisher.

## Supplementary Material

Supplementary Figure 1 Staining for RPE-marker RPE-65 (green) and CRX (cone-rod homeodomain transcription factor, marker for photoreceptor progenitors and later cone bipolar cells). **(A,B)** Transplant #1, 176 dps (age 228 days). The space in the section and the lifting of the parylene is created by a processing artifact. The transplant RPE is stained for RPE-65.

## Abbreviations

AMC: age matched control
AMD: age-related macular degeneration
BM: Bruch’s membrane
cd/m^2^: candela per meter square
CRALBP: cellular retinaldehyde binding protein
CRX: cone-rod homeobox gene
DAB: diaminobenzidine
DAPI: 4,6-diamidino-2-phenylindole
dps: days post-surgery
FACS: fluorescent activated cell sorting
FDA: Food and Drug Administration
GFP: green fluorescent protein
H: host (recipient)
H&E: hematoxylin–eosin
hESCs: human embryonic stem cell(s)
IFA: immunofluorescence
INL: inner nuclear layer
IPL: inner plexiform layer
LE: left eye
ms: millisecond
MVG: medium viscosity
OCT: optical coherence tomography
OKT: optokinetic testing
ONL: outer nuclear layer
OS: outer segments of photoreceptors
PBS: phosphate-buffered saline
PR: photoreceptor
p.s.: post-surgery
RCS: Royal College of Surgeons
RD: retinal degeneration
RE: right eye
RO: retina organoid
RP: retinitis pigmentosa
RPE: retinal pigment epithelium
SC: superior colliculus
SD-OCT: spectral domain optical coherence tomography
T: transplant
UCI, University of California: Irvine
USC, University of Southern California: Los Angeles.
