# Supplementary figures and images for: Co-grafts of Human Embryonic Stem Cell Derived Retina Organoids and Retinal Pigment Epithelium for Retinal Reconstruction in Immunodeficient Retinal Degenerate Royal College of Surgeons Rats
Source: Front Neurosci. 2021 Oct 26;15:752958. doi: 10.3389/fnins.2021.752958 (PMC8576198; doi:10.3389/fnins.2021.752958)

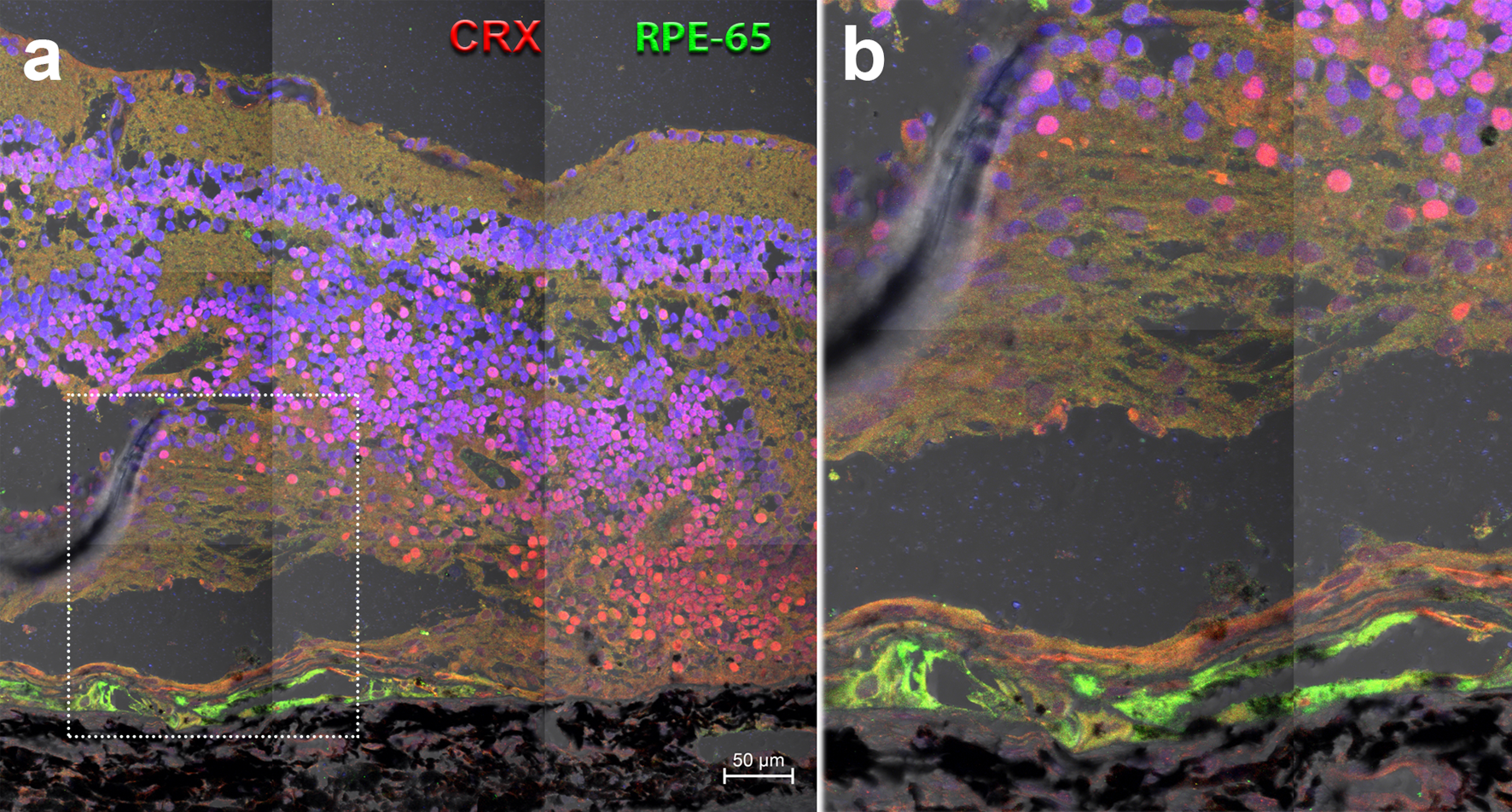

Supplement: Supplementary Figure 1 — Staining for RPE-marker RPE-65 (green) and CRX (cone-rod homeodomain transcription factor, marker for photoreceptor progenitors and later cone bipolar cells). (A,B) Transplant #1, 176 dps (age 228 days). The space in the section and the lifting of the parylene is created by a processing artifact. The transplant RPE is stained for RPE-65. [file Image_1.TIF]
